# How Modeling Can Reconcile Apparently Discrepant Experimental Results: The Case of Pacemaking in Dopaminergic Neurons

**DOI:** 10.1371/journal.pcbi.1002050

**Published:** 2011-05-26

**Authors:** Guillaume Drion, Laurent Massotte, Rodolphe Sepulchre, Vincent Seutin

**Affiliations:** 1 Laboratory of Pharmacology and GIGA Neurosciences, University of Liège, Liège, Belgium; 2 Department of Electrical Engineering and Computer Science, University of Liège, Liège, Belgium; University of Freiburg, United States of America

## Abstract

Midbrain dopaminergic neurons are endowed with endogenous slow pacemaking properties. In recent years, many different groups have studied the basis for this phenomenon, often with conflicting conclusions. In particular, the role of a slowly-inactivating L-type calcium channel in the depolarizing phase between spikes is controversial, and the analysis of slow oscillatory potential (SOP) recordings during the blockade of sodium channels has led to conflicting conclusions. Based on a minimal model of a dopaminergic neuron, our analysis suggests that the same experimental protocol may lead to drastically different observations in almost identical neurons. For example, complete L-type calcium channel blockade eliminates spontaneous firing or has almost no effect in two neurons differing by less than 1% in their maximal sodium conductance. The same prediction can be reproduced in a state of the art detailed model of a dopaminergic neuron. Some of these predictions are confirmed experimentally using single-cell recordings in brain slices. Our minimal model exhibits SOPs when sodium channels are blocked, these SOPs being uncorrelated with the spiking activity, as has been shown experimentally. We also show that block of a specific conductance (in this case, the SK conductance) can have a different effect on these two oscillatory behaviors (pacemaking and SOPs), despite the fact that they have the same initiating mechanism. These results highlight the fact that computational approaches, besides their well known confirmatory and predictive interests in neurophysiology, may also be useful to resolve apparent discrepancies between experimental results.

## Author Summary

Dopamine is a neurotransmitter which plays important roles in the control of voluntary movement, motivation and reward, attention, and learning. Dysfunction of midbrain dopaminergic systems is involved in various diseases such as Parkinson's disease, schizophrenia and drug abuse. This underlines the importance of a tight regulation of dopamine levels in the brain. At the cellular level, the release of dopamine is directly correlated to the type of electrical activity (the firing pattern) of nerve cells that produce it, the so-called “dopaminergic neurons”. Therefore, an in depth understanding of the mechanisms underlying the electrical behavior of dopaminergic neurons is of critical importance to find new strategies for the treatment of diseases that result from dysfunction of this system.

## Introduction

Midbrain dopaminergic (DA) neurons sustain important physiological functions such as control of movement [1] and signalling of positive error in reward prediction [2]. A dysfunction of the DA system is implicated in the pathophysiology of Parkinsons disease, schizophrenia, and drug abuse [3]. Under physiological conditions, DA neurons can switch between three distinct modes: tonic (pacemaker), irregular, and burst firing [4], [5].

The nature of the channels involved in the low frequency pacemaking of DA neurons is still strongly discussed. Indeed, whereas many studies have shown that L-type calcium channels are critical for this spontaneous activity, others, including ours, have observed little effect of a blockade of these channels on this firing pattern (see Table 1). Therefore, the respective contribution of calcium and sodium channels in pacemaking remains unclear. On the other hand, it is commonly accepted that low-frequency spontaneous firing requires oscillations in the cytoplasmic free calcium concentration [6], [7].

**Table 1:** Effect of manipulations that block voltage-dependent 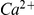 channels on the pacemaking of midbrain DA neurons *ex vivo* or *in vitro*.

Reference	Nature of the preparation	Agent used	Observed effect
Nedergaard et al., 1993	Slices from adult guinea-pigs, SNc, intracellular recordings.	nifedipine ( 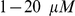 )	Cessation of firing at undisclosed concentration.
Mercuri et al., 1994	Slices from adult Wistar rats, SNc and lateral VTA, intracellular recordings.	nifedipine and nimodipine ( 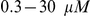 )	Decrease in the firing rate of about 50 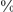 with 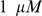 of both drugs.Cessation of firing with 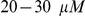 of both drugs.
Puopolo et al., 2007	Acutely dissociated neurons from the SNc of juvenile (16 day-old) mice, whole cell recordings.	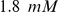 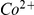 in replacement of 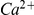 nimodipine ( 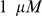 ) 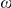 -aga-IVA ( 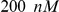 )	Cessation of firing in all neurons (17/17).Firing rate decreased in 9/17 neurons.Firing rate decreased in 10/14 neurons.
Chan et al., 2007	Slices from juvenile mice (younger than P21), SNc, cell-attached and whole-cell recordings.Slices from young adult mice (older than P28), SNc, cell-attached and whole cell recordings.	isradipine ( 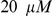 ) and nimodipine ( 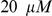 )isradipine ( 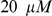 ) and nimodipine ( 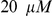 )	“Firing largely unaffected” (but firing reduced by an 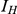 blocker).Cessation of firing in all neurons (15/15): “plastic” phenomenon in “several” neurons (firing resumes during block 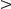 1 hour in some neurons).
Guzman et al., 2009	Slices from both juvenile and young adult mice, SNc, cell-attached and whole cell recordings	isradipine ( 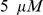 )	Firing unaffected.
Putzier et al., 2009	Slices from juvenile rats (younger than P21), SNc, whole cell recordings	nimodipine ( 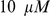 )	Cessation of firing.
Khaliq and Bean, 2010	Slices from both juvenile and young adult mice, medial VTA, whole cell recordings	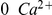 , 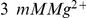	Firing increased three-fold.
Seutin et al., unpublished	Slices from adult ( 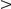 6 week-old) rats, SNc, extracellular recordings)	nifedipine ( 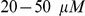 )nimodipine ( 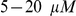 )	Firing unaffected (N = 5).Variable effects, no clear trend (N = 5).

SNc: substantia nigra, pars compacta; VTA : ventral tegmental area. Rodents are classified as juvenile (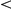 P21), young adults (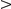 P28) or adult (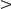 6 weeks).

In the presence of the sodium channel blocker tetrodotoxin (TTX), DA neurons also exhibit slow oscillatory potentials (SOPs) [8], [9], which have been shown to be sustained by L-type calcium channels [10]. Guzman et al. recently observed that SOPs and spikes are not correlated in rate and regularity, from what they concluded that pacemaking and SOPs are driven by different mechanisms [10]. This conclusion is used to support the hypothesis that L-type calcium channels would not strongly contribute to pacemaking in DA neurons, which is in opposition with many experimental results [6], [11] (Table 1). An additional controversy results from the fact that the block of SK channels prolongs depolarizing plateaus under sodium channel inhibition, whereas it only slightly affects the firing rate when the neurons fire action potentials.

In this paper, we use a mathematical analysis to extract the mechanisms underlying the spontaneous activity of DA neurons. For this purpose, we develop a minimal model of a DA neuron, in which we include the minimal set of conductances that are able to reproduce the firing patterns exhibited by these cells (see below). This minimal model is able to exhibit pacemaker firing in the absence of synaptic afferents, and to switch from a low frequency single-spike firing to bursting when SK channels are blocked in the presence of excitatory inputs, as reported experimentally [12], [13]. Moreover, SOPs are present during the inhibition of sodium channels, and the effect of a SK channel blockade observed experimentally is reproduced in the model. In order to validate our analyses of the minimal model, we also test its predictions on a published detailed model of these neurons [14], [15].

The main conclusion of our analysis is that pacemaker firing in DA neurons is sustained by the cooperation of sodium and L-type calcium channels (and more modestly N-type or P/Q-type calcium channels [6]), whereas variations of the intracellular calcium concentration play a major role in the rate of this spontaneous firing pattern. On the basis of this mechanism, we identify potential causes for the experimental discrepancies mentioned above, using our minimal model, as well as the detailed model. We observe that neurons only differing by less than 1% in their maximal sodium conductance react oppositely to a blockade of L-type calcium channels. Experiments performed in rat brain slices confirm that L-type calcium and sodium channels cooperate to generate pacemaking in these neurons.

As a secondary conclusion, our model shows that, even though the initiation of SOPs and spikes is sustained by the same mechanism, these oscillatory patterns are not correlated, which is in agreement with experimental results [10]. We show that this absence of correlation is due to different mechanisms of depolarizing phase termination in the two oscillatory behaviors, as well as different kinetics of calcium entry (resp. exit) during depolarizing phases (resp. hyperpolarizing phases). These results show that the lack of correlation between pacemaking and SOPs does not exclude a shared mechanism.

## Results

### A minimal model to capture the pacemaking mechanism of dopaminergic neurons

**Figure 1:**
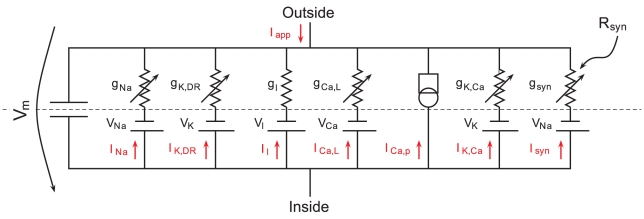
Equivalent circuit diagram of the model. The model is composed of one compartment containing the conductances shown, in parallel with a membrane capacitance.

In order to extract the essential mechanisms of pacemaking of DA neurons, we developed a minimal model endowed with the minimal set of conductances which is necessary to reproduce the firing patterns of these cells. The conductances present in the model are shown in Fig. 1 and the corresponding equations are detailed in the Methods section. In parallel with the membrane capacitance and a leak current, the model is composed of sodium channels and delayed-rectifier potassium channels for the generation of action potentials (fast dynamics). Calcium enters through L-type calcium channels endowed with calcium dependent inactivation (CDI) [16], [17], whereas calcium extrusion is carried out by calcium pumps. In addition, small conductance calcium-activated potassium (SK) channels are present. Finally, an excitatory synaptic current is added to test the effect of noise of variable amplitude on the firing of the modeled cell (small amplitude noise to mimic *in vitro*-like conditions and high amplitude noise to mimic *in vivo*-like conditions). In the presence of SK channels, the minimal model does not produce high firing frequencies in response to a step of applied depolarizing current but it does so in response to a high amplitude synaptic input. Calcium dynamics is well-known to be essential to classical models of bursting neurons [18], hence the combination of a calcium-regulated inward current (carried by L-type calcium channels) and a calcium-regulated outward current (carried by calcium pumps). In our model, however, the neuron produces only one action potential per oscillation cycle of 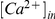, which results in low-frequency single-spike firing rather than bursting in *in vitro*-like conditions (Drion et. al, unpublished).

**Figure 2:**
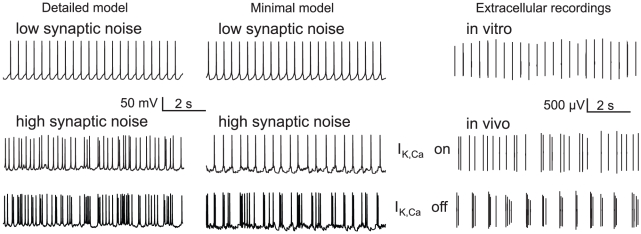
Comparison of the behavior of the detailed model (left) and of the minimal model (middle) in *in vitro* and *in vivo*-like conditions with experimental data obtained from dopaminergic neurons (right). In each case, the neuron fires regularly in single spikes *in vitro*, and an inhibition of calcium-activated potassium channels induces burst firing *in vivo*. Experimental data are from Seutin (unpublished)(upper panel) and Drion (unpublished)(lower panel).

The proposed minimal model is able to reproduce the firing patterns exhibited by DA neurons, namely pacemaker firing *in vitro*, irregular single-spike and burst firing *in vivo*, or be in a hyperpolarized state (Fig. 2). As it has been shown experimentally, the switch between irregular single-spike firing and bursting can be induced by a blockade of small conductance calcium-activated potassium (SK) channels [12], [13]. In the remainder of this paper, we will only discuss the mechanisms of spontaneous firing *in vitro* (i.e. when the neuron is submitted to small amplitude noise). Also, in order to verify that our simplified model correctly captures the firing mechanisms of DA neurons, we systematically test the predictions of the minimal model against simulations performed with the detailed model (see the equivalent circuit in [14]). This much more complex model exhibits the majority of the channels which have been reported to be present in DA neurons experimentally and takes the general architecture of the cell into account.

The frequency of the spontaneous activity is limited by calcium influx. Indeed, a rise of the cytoplasmic calcium concentration strongly reduces the excitability of the cell. Therefore, to ensure a calcium entry (resp. exit) during the spike generation (resp. between two successive spikes), one or several types of calcium channels (resp. calcium pumps) must be present. Moreover, in order to generate a spontaneous activity even in the absence of calcium-activated potassium channels (as is observed in DA neurons [19]), the calcium dynamics must be regulated by calcium, e.g. through the presence of calcium-inactivated calcium channels. Under these two conditions, the cell is able to generate a low-frequency spontaneous activity as well as fast firing when submitted to excitatory synaptic inputs, with a very different firing rate between these two firing patterns (0.5 to 5 Hz and more than 15 Hz, respectively, see Fig. 2).

### Experiments on neurons with very similar channel build-ups may lead to contradictory observations

**Figure 3:**
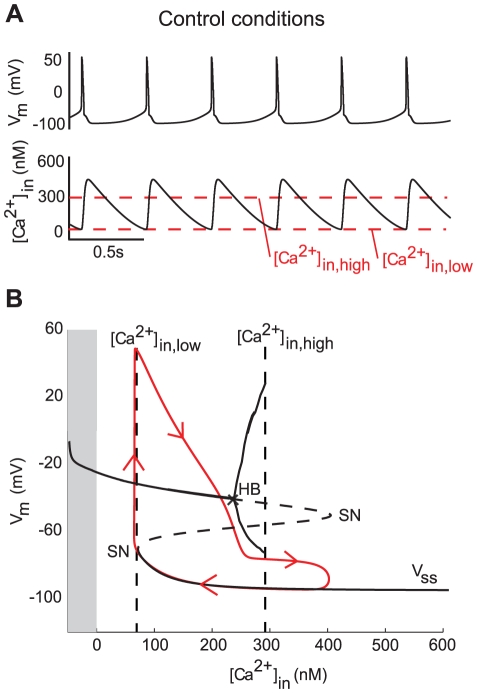
Analysis of the spontaneous activity of the minimal model. (**A**) Variations of the membrane potential (top) and the intracellular calcium concentration (bottom) over time. (**B**) Sketch of the bifurcation diagram of the minimal model, with 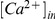 as the bifurcation parameter. The gray part corresponds to negative values of 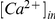, which is non physiological. 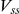 denotes the steady-state curve for each value of the bifurcation parameters. The dotted part of 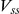 shows its unstable part. HB denotes a Hopf bifurcation and SN a saddle-node bifurcation. The trajectory of the membrane potential is plotted in red.

In the absence of synaptic inputs, DA neurons fire spontaneously in a very regular manner [19], [20]. The mechanisms underlying this pacemaker activity are still strongly discussed, experimental results being contradictory (Table 1). However, it is commonly accepted that pacemaking of DA neurons requires calcium oscillations and that SK channels are not critical to sustain this firing pattern [19]. Fig. 3 illustrates the mechanisms involved in pacemaker firing in the minimal model. As it has been demonstrated experimentally [10], the spontaneous activity is synchronized with calcium oscillations. Namely, each action potential is generated when the intracellular calcium concentration reaches a constant minimal value (Fig. 3A). This is consistent with experimental results [9], [10].

The mechanisms involved in the pacemaker activity of the minimal model can be fully understood using bifurcation analysis. The bifurcation diagram shown in Fig. 3B illustrates how the spike generation is governed by the intracellular calcium concentration, the latter being the bifurcation parameter. The diagram defines three distinct ranges of intracellular calcium concentration : a low range where the only stable steady-state is depolarization block (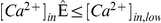); a high range where the only stable steady-state is hyperpolarization (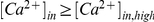); and an intermediate bistable range where a limit cycle may coexist with the two stable steady-states (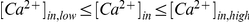). The thresholds separating these zones are mainly dependent on the regulation of calcium channels and calcium pumps by the intracellular calcium concentration. Thus, a rise of the intracellular calcium concentration induces an inactivation of the L-type calcium channels [16], [17], which reduces the amount of inward (i.e. depolarizing) current, and an activation of calcium pumps, which increases the amount of outward (i.e. repolarizing) current [21]. As a consequence, the excitability of the cell decreases with the intracellular calcium concentration. A variation of the intracellular calcium concentration that exceeds the high threshold will induce a switch from firing to a hyperpolarized state. Therefore, the high threshold, which is defined as the value of 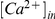 at which the stable limit cycle disappears, defines the maximal possible intracellular calcium concentration which is compatible with firing.

The low threshold in Fig. 3 defines the value of intracellular calcium concentration at which an action potential is spontaneously generated. As a consequence, the current which initiates the depolarization at this point is the critical one for pacemaking. For instance, for the set of parameter values used in Fig. 3, action potentials are generated by an opening of L-type calcium channels, and a complete blockade of these channels prevents firing. But if we use other sets of values for the conductance of sodium and L-type calcium channels, the mechanism of spontaneous initiation of spikes can vary. This suggests a strong cooperation between sodium and L-type calcium channels to drive the pacemaker activity of DA neurons, as suggested by Guzman et. al [10].

**Figure 4:**
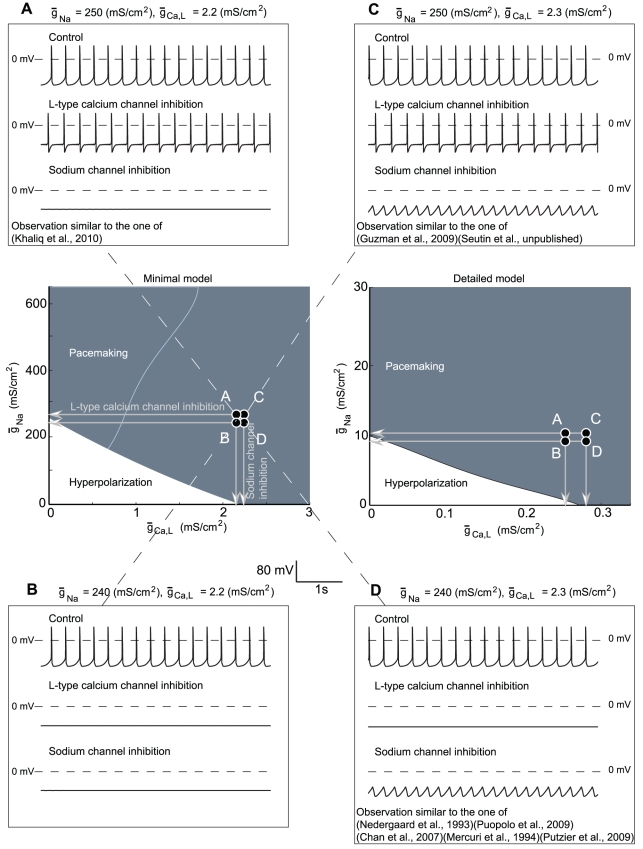
Cooperation between sodium and calcium channels in the generation of spontaneous activity in the minimal model and in the detailed model. The center panels show the type of pacemaker activity according to the value of sodium and L-type calcium conductances. The white zone represents the couples of conductances which result in a hyperpolarized state of the cell and the dark blue zone accounts for pacemaking. Each insert shows the behavior of the model in control condition and during a blockade of L-type calcium channels or sodium channels for a particular set of conductances. The pacemaker behavior of the model strongly relies on the values of both the sodium and the L-type calcium conductances.

The important consequences of this cooperation are illustrated in Fig. 4. The threshold separating a hyperpolarized state (white) from pacemaking (blue) is defined by an almost linear combination of the L-type calcium conductance 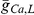 and the sodium conductance 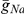. Moreover, when the conductance of one channel type is above a threshold value, these channels are sufficient to drive a spontaneous activity during blockade of the others. As a consequence, very similar neurons which would have minimal differences in their sodium or L-type calcium channel density may exhibit very different responses to experimental manipulations that shut off one of the two conductances, as illustrated in the various inserts of Fig. 4.

Panels **A** to **D** of Fig. 4 show the behavior of the minimal model in control conditions and during blockade of L-type calcium channels or sodium channels for a particular set of conductances. The blockade is modeled by setting the conductance to zero. Only the sodium and L-type calcium conductances slightly differ in the four represented situations. Note that the electrical behavior of each modeled neuron in control conditions is almost similar: the differences in parameter values are very small, and the mechanisms that underly their spontaneous activity are similar. However, these neurons react very differently to the blockade of one conductance. In the case of neuron **D** (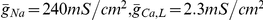), L-type calcium current inhibition completely inhibits the spontaneous activity of the cell, whereas an oscillatory behavior remains after a sodium current inhibition. On the basis of these experimental-like scenarii, we would conclude that the pacemaker activity of neuron **D** is driven by L-type calcium channels. If we examine neuron **A** (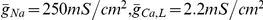), L-type calcium current inhibition barely affects the firing rate and pattern of the cell, whereas a sodium current inhibition induces a hyperpolarization of the membrane. These observations would therefore lead to the opposite conclusion, namely that the pacemaker activity of neuron **A** is driven by sodium channels. As a consequence, the results of the two experiments would be contradictory, despite the great similarity of the neurons. More generally, Fig. 4 illustrates the fact that very similar neurons may produce drastically different responses to the same experimental manipulation. Remarkably, the four distinct behaviors exhibited by almost identical neurons in the minimal model can be exactly reproduced in the detailed model (see details in Supplementary Fig. S1).

### Experimental confirmation that sodium and L-type calcium channels cooperate to generate spontaneous pacemaking in SNc DA neurons

In order to confirm that the spontaneous initiation of spikes in DA neurons is mainly sustained by the cooperation between sodium and L-type calcium channels, we performed extracellular recordings (additional to those reported in Tab. 1) of these neurons in slices from adult rats containing the substantia nigra pars compacta. A potential advantage of this recording method is that it does not disrupt the contents of the neuron, contrary to conventional patch-clamp recordings. For these experiments, we superfused the slices with blockers of synaptic transmission (10 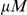 CNQX, 1 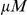 MK801, 10 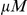 SR95531, 1 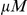 sulpiride and 1 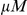 CGP55845, which block AMPA, NMDA, 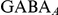, D2 and 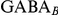 receptors, respectively), in order to isolate the neurons from their afferences. Control experiments showed that application of the synaptic blockers alone induced a small increase in firing rate which was stable for at least one hour (n = 4, supplementary Fig. S2).

**Figure 5:**
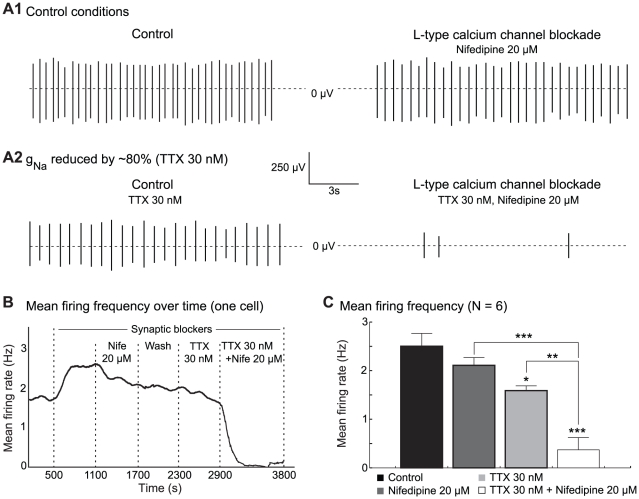
Effect of sodium and L-type calcium channel blockade on the firing of SNc dopaminergic neurons *in vitro*. (**A1**) Extracellular recording of a DA cell in control conditions (left) and after application of 20 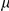
M nifedipine (right). (**A2**) Same as (**A1**) after a 80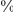 reduction of the sodium conductance by the superfusion of 30 nM TTX. (**B**) Evolution of the mean firing rate (samples of 2 minutes) of a DA cell over time. (**C**) Mean firing frequency (N = 6) for each condition (mean 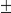 sem). A simultaneous application of TTX and nifedipine affects the firing of the cells more strongly, as compared to the application of either of the two compounds alone. All experiments were performed in the presence of blockers of synaptic transmission. Note that the superfusion of the blockers produces an excitation of the neurons, which can be attributed to the block of inhibitory D2 autoreceptors. *P
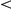
0.05, **P
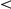
0.01, ***P
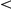
0.001.

We next tested the effect of 20 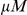 nifedipine (a L-type calcium channel blocker), 30 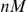 TTX (a sodium channel blocker), as well as their simultaneous application on the firing rate of DA cells, respectively. This concentration of TTX was used because it had been shown to block a major fraction (about 80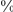) of the somatic sodium conductance [22], but did not abolish action potentials. The precise experimental protocol is shown graphically in Fig. 5B and is described in detail in the Methods section.

These recordings were performed on eleven neurons. In one case, nifedipine completely inhibited the spontaneous activity of the cell. In four other cells, nifedipine produced little effect, whereas TTX completely suppressed the firing (not shown). In the six other neurons, coapplication of nifedipine and TTX inhibited the firing to a greater extent than either agent alone. Indeed, an ANOVA test showed that the firing rate of the neurons was different in the four experimental conditions (synaptic blockers alone, +nifedipine, +TTX, +TTX and nifedipine, F[3], [20] = 21.12, p = 0.000002). The application of nifedipine alone did not significantly affect the firing rate of these cells (from 2.51
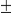
0.26 Hz to 2.11
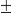
0.16 Hz, mean 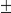 s.e.m., p
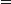
0.52, Tukey's post hoc test) (Fig. 5). The application of TTX alone significantly but only partially decreased the firing rate of the cells to 1.59
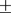
0.10 Hz (p
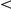
0.05), and reduced the amplitude of spikes. The latter effect is in agreement with the fact that the maximal sodium conductance is quite reduced. The simultaneous application of TTX and nifedipine reduced the firing rate of the cells to 0.37
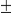
0.25 Hz (p
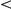
0.001), and this reduction was significantly larger than the effect of either agent alone (p
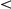
0.001 vs nifedipine, p
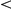
0.01 vs TTX). Moreover, the simultaneous pharmacological block of sodium (80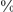) and L-type calcium channels almost completely eliminated the spontaneous firing in 5 of these 6 cells (frequency 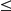
0.4 Hz). In summary, our experiments confirm that the degree of cooperation between the two currents is highly variable from neuron to neuron, even in a fixed experimental protocol.

### Pacemaking can be driven by different cooperating currents

The fact that L-type calcium channels and sodium channels can cooperatively drive the pacemaker activity of DA neurons can be explained by comparing the I-V curves of the two currents (Supplementary Fig. S3A). Indeed, Putzier et al. recently showed that the only critical parameter of the L-type calcium current for pacemaking is the value of its half-activation potential (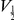), which should not be too negative [11]. Moreover, they showed that artificial NMDA receptors that would have a similar half-activation potential would also induce sustained firing. The half-activation potentials of sodium and L-type calcium channels are very similar (Supplementary Fig. S3A), which explains why they can cooperate in the pacemaking generation.

**Figure 6:**
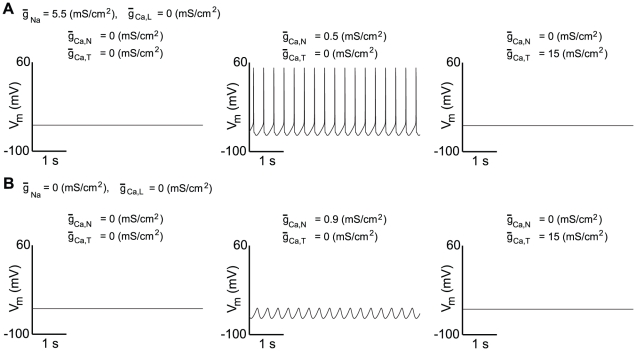
Ability of a N-type, but not of a T-type calcium current, to drive pacemaker activity in the absence of all other calcium channels in the detailed model. (**A**) Variations of the membrane potential of the modeled neuron over time when all calcium currents are blocked, in the presence of N-type calcium channels and in the presence of T-type calcium channels, from left to right, respectively. (**B**) Same as (**A**), but during an inhibition of sodium channels. N-type calcium channels in sufficient density are able to generate an oscillatory behavior, contrary to T-type calcium channels.

This similarity is observed in the detailed model as well (Supplementary Fig. S3B). If we compare the I-V curves of the other calcium currents, it is clear that, in this model, N-type calcium channels have a 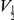 similar to the one of sodium and L-type calcium channels, whereas 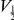 of T-type calcium channels is much more negative. On the basis of these observations, the previous analysis suggests that N-type calcium channels should be able to induce pacemaking if their density is high enough whereas T-type channels, which have a significant more negative 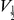, should not. We test this hypothesis in Fig. 6 (where we reduce the sodium conductance to reduce the contribution of sodium channels to the pacemaking) using the detailed model. As predicted, a N-type calcium current of sufficient amplitude is able to drive a low-frequency pacemaker activity, whereas a T-type calcium current is not, even with a very high maximal conductance value. Moreover, N-type channels are able to induce SOPs when sodium channels are blocked, as L-type channels do (Fig. 4C and D). These simulations are therefore in agreement with the prediction that whatever the nature of the depolarizing current, the only parameter which is critical is its voltage dependence, and more precisely its half-activation potential [11].

### The common mechanism of pacemaking and slow oscillatory potentials does not imply that these oscillatory activities are correlated or are affected in the same way by the same experimental manipulation

Our minimal model also sheds light on the mechanisms of, and related controversies on, slow oscillatory potentials (SOPs). Indeed, it has experimentally been shown that SNc DA neurons exhibit SOPs during application of TTX *in vitro* [8], [9], and that these SOPs are driven by L-type calcium channels [10]. Recently, it has been shown that pacemaking and SOPs are uncorrelated [10], which led to the conclusion that these two oscillatory behaviors are driven by different mechanisms, thus rejecting L-type calcium channels for pacemaking.

In addition, it has been reported that block of the SK current on TTX-treated DA neurons strongly affects the shape of SOPs. Namely, this manipulation significantly increases the duration of the depolarizing and hyperpolarizing phases [9]. This observation led to the hypothesis that, in control condition, this inhibition might induce burst firing in DA neurons. However, experiments have invalidated this intuitive suggestion, SK channel blockade only inducing irregularities in the firing of these cells, but not bursting (at least not reproducibly) [19]. These contradictory observations can be explained through an analysis of the behavior of the minimal model in configurations that mimic these experiments.

**Figure 7:**
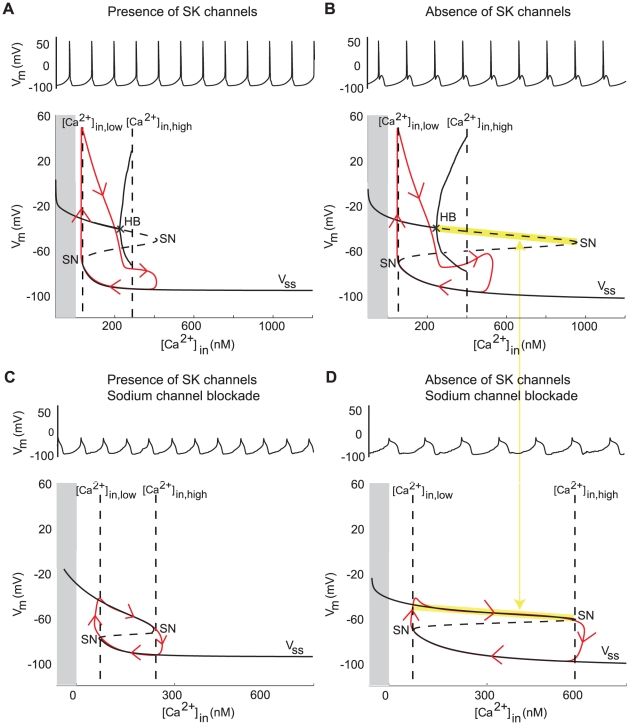
Analysis of the effect of SK channel blockade on the spontaneous activity of the minimal model. (**A** and **B**) Variations of the membrane potential over time (top) and sketch of the bifurcation diagram of the minimal model (bottom, with 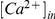 as the bifurcation parameter) in the presence and in the absence of SK channels. The grey part corresponds to negative values of 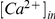, which are non physiological. 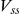 denotes the steady-state curve for each value of the bifurcation parameters. The dotted part of 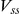 shows its unstable part. HB denotes a Hopf bifurcation and SN denotes a saddle-node bifurcation. Trajectories of the membrane potential are plotted in red. (**C** and **D**) Same as (**A** and **B**) but when sodium channels are blocked. The frequency of SOPs is almost halved, whereas the frequency of spikes is barely affected. This simulations were performed in the absence of noise, which would induce irregularities in the absence of SK channels.

For a sufficiently high value of 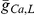 (compare Fig. 4C,D and Fig. 4A,B), SOPs are also observed after blockade of the sodium current in the minimal model (Fig. 7C). As in the case of pacemaking, these SOPs are synchronized with the calcium oscillations (supplementary Fig. S4). Fig. 7 shows how sodium channel blockade affects the bifurcation diagram in the presence and in the absence of SK channels. As illustrated in the figure, sodium channel blockade has no effect on the low threshold, which implies that a same mechanism initiates both spikes and SOPs in the minimal model.

In contrast, sodium channel blockade has a critical impact on the high threshold. Indeed, the Hopf bifurcation which defines the high threshold in pacemaking vanishes when sodium channels are blocked. As a consequence, the high threshold of SOPs is defined at the right saddle-node bifurcation, which is masked by the Hopf bifurcation in control conditions. This implies that, whereas the depolarization mechanism is identical in spikes and SOPs, the repolarization mechanisms are different.

This difference has significant consequences on the model behavior, all consistent with experimental data:

- SK channel blockade has little effect on the left saddle-node bifurcation but a dramatic effect on the right one (compare Fig. 7A,C and Fig. 7B,D). This effect is masked in normal conditions because of the Hopf bifurcation. As a consequence, inhibition of the SK current barely affects the pacemaker firing in normal conditions (Fig. 7A,B) but strongly affects SOP frequency when sodium channels are blocked (Fig. 7C,D). These predictions are in agreement with experimental data [9], [19].
- There is no reason to expect a strong correlation between spikes and SOPs.

**Figure 8:**
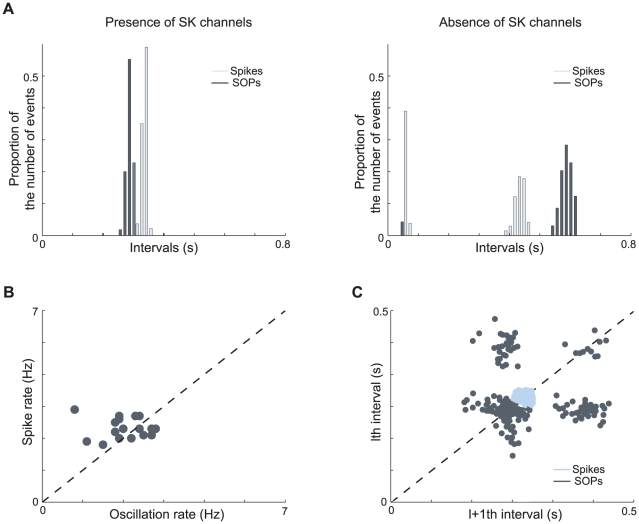
Comparison of the pacemaking (“spikes”) and slow oscillatory potentials (“SOPs”) in the minimal model. Note that the simulations have been performed using *in vitro*-like conditions (low amplitude noise). (**A**) Interspike interval histograms (ISIh's) of a set of modeled cells in the presence (left) and in the absence (right) of SK channels. The light blue bars account for spikes in control conditions and the dark blue bars for SOP oscillations during sodium channel blockade. (**B**) Comparison of the rate of spikes and SOPs for different values of 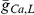 and 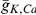. (**C**) Plot of successive spike intervals (light blue) or successive SOP intervals (dark blue) of a set of modeled cells. Although they are driven by the same mechanisms, spikes and SOPs are not correlated. Moreover, SOPs are more sensitive to the noise.

Fig. 8A shows that the interevent interval histograms of pacemaking (in light blue) and SOPs (in dark blue) do not match either in the presence (Fig. 8A, left) or in the absence (Fig. 8A, right) of SK channels. Moreover, depending on the parameters of the model, spike rate can be lower or higher than SOP oscillation rate (Fig. 8B), as observed experimentally [10]. In Fig. 8, the parameters that are varied are the conductances of L-type calcium channels and SK channels. The main origin of this lack of correlation is that the quantity and kinetics of calcium entry taking place during an action potential strongly differs from the one taking place during the depolarized phase of SOPs. This affects the rate of their respective oscillations (Supplementary Fig. S5).

In addition, Guzman et. al recently showed that spikes are much more regular than SOPs in DA neurons [10]. In its low-noise configuration (representing the *in vitro* condition), our model exhibits similar results (Fig. 8C, which can be compared to Fig. 3e of [10]). Indeed, we found that SOPs are much more sensitive to noise than spikes in the presence of SK channels, mainly because SOP oscillations induce less calcium entry than spikes and because the kinetics of calcium entry is slower (supplementary Fig. S5).

These results show that a lack of correlation between spikes and SOPs does not necessarily imply that the generating mechanism of these two oscillatory behaviors is different. Therefore, this experimental observation may not be used to dismiss a role of L-type calcium channels in the generation of spikes in DA neurons.

## Discussion

### The generation of spikes during low-frequency pacemaker firing mainly relies on the cooperation between sodium and L-type calcium channels, whereas its rhythm follows the variations in 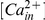


In spite of many experimental studies, the precise mechanisms underlying the spontaneous initiation of spikes in DA neurons are still largely debated in the literature. Using our minimal model as well as a detailed model of a DA neuron, we extracted two critical parameters for the low frequency spontaneous firing.

Firstly, low-frequency single-spike firing and high-frequency intra-bursts firing have to be sustained by two dynamics operating on different time scales. Fast firing is limited by the refractory period of action potentials, which is fixed by the kinetics of voltage-gated channels. On the other hand, the dynamics that are the most likely to limit the rate of low-frequency firing are the variations of the intracellular calcium concentration. Indeed, an accumulation of calcium in the cytoplasm strongly reduces the excitability of the cells, through the inactivation of depolarizing currents (i.e. L-type calcium currents) and the activation of hyperpolarizing currents (i.e. calcium pumps and SK channels). This is in agreement with experimental data, which show that replacement of calcium with either cobalt [6] or magnesium [7] strongly affects pacemaking.

Secondly, the spontaneous initiation of action potentials in DA neurons is the result of the cooperation between various depolarizing currents. In agreement with the experimental results of Putzier et al. [11], we found in the detailed model that any depolarizing current having a half-activation potential less negative than 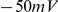 (voltage-dependant sodium channels, L-type and N-type calcium channels) may play a role in this initiation. Their relative contribution, as well as the robustness of pacemaking, depend on the respective density of each channel type. Therefore, the fact that the selective blockade of a particular channel does not completely disrupt pacemaking does not mean that these channels are not involved in physiological pacemaking. This might be an important note of caution for experimentalists. Moreover, we confirmed experimentally that L-type calcium and sodium channels do indeed cooperate to generate pacemaking with a degree of cooperation that is highly variable. This precise observation has never been made previously.

### Experimental protocols are not robust to physiologically plausible variability

The most contradictory experimental results obtained on DA neurons are probably those concerning the role of L-type calcium channels in the spontaneous initiation of spikes *in vitro* (Table 1). Using very similar modeled neurons that differ by less than 1% in one conductance parameter (all remaining parameters being identical), we were able to reproduce these contradictory results both in our minimal model and in a detailed model of DA neurons.

Such subtle differences in conductance parameters are quite likely to occur in various experimental conditions. For example, it has recently been shown that there are quantitative differences between DA neurons from the SNc and the VTA in terms of density of these conductances [6], [7]. It was proposed that sodium channels play a major role in the spike generation of VTA DA cells, whereas calcium channels are predominant in SNc DA neurons. Among other findings, replacement of calcium with cobalt in SNc neurons completely inhibits the firing, whereas replacement of calcium with magnesium in VTA neurons increases the firing rate [6], [7]. Both these effects are also observed in the minimal and detailed models with slightly different maximal sodium conductances (supplementary Fig. S6).

A second source of contradictory experimental results might be the difference between the preparations that are used in different laboratories. For instance, in the case of DA neurons, in which the initial segment is often remote from the soma [23], it is clear that the total sodium current will be smaller in acutely dissociated neurons than in neurons recorded in the slice preparation. In terms of the model that we have developed, this probably means that the small conductance variations illustrated in Fig. 4 could result from minor experimental variations such as dissociated neurons vs neurons recorded in slices, as well as variable developmental stages of the animals.

### The lack of correlation between spikes and SOPs is compatible with a same generating mechanism

SOPs exhibited by DA neurons during blockade of sodium channels have been largely studied [8]–[10] and compared to the spontaneous spiking activity. Moreover, it has been recently shown that the two oscillatory patterns are not correlated [10], a phenomenon that is also observed in our model. It is tempting to conclude from this observation that their underlying mechanisms are different. However, our analysis shows that the generation mechanisms are actually the same. Moreover, Fig. 7 clearly illustrates that the different effect of SK channel blockade on pacemaking and SOPs simply arises from the fact that the high intracellular calcium concentration threshold only slightly changes when sodium channels are present, whereas a more than two fold change in this high threshold occurs when sodium channels are blocked.

### Added value of a minimal model in the analysis of DA neuron electrophysiology

DA neuron electrophysiology has been modeled by several groups [14], [24]–[29]. Most of these models were elaborated in order to reproduce experimental observations. Although they clearly succeed at making detailed predictions, their complexity may prevent a detailed analysis of the mechanisms underlying pacemaking in these neurons. On the basis of these models, we attempted to develop a minimal model containing only the most critical conductances needed to reproduce firing patterns of DA neurons. This model allowed us to explain some discrepancies found in the literature on the mechanisms underlying pacemaking of these cells. Importantly, we were able to confirm our conclusions on one detailed model [14].

There are some conceptual differences between our minimal model and some earlier models. For example, the calcium current included in the Wilson and Callaway model [26] does not include any calcium-induced inactivation, whereas ours does. We included inactivation because recent experiments have demonstrated that the L-type channels expressed by DA neurons belong to the 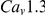 class [30] and this channel subtype is known to undergo calcium-induced inactivation in expression systems [17]. Clearly, this point has to be carefully investigated in future experiments on DA neurons. It should be pointed out, however, that inactivation of L-type channels is not fundamental for the results that we obtained in our model. Indeed, the major role of this phenomenon is to decrease the excitability of the cell when intracellular calcium rises after the action potential. This decrease could be induced by other mechanisms, such as activation of a potassium ERG-type current [29].

One limitation of our minimal model is that it is essentially qualitative and does not take into account the very specifics of DA neurons. However, because of its generality, our model could be a starting point to analyze similar firing patterns of a number of other cell types. Finally, our analysis demonstrates the value of a simplified model to reconcile apparently contradictory experimental observations.

## Materials and Methods

### Ethics statement

All procedures were carried out in accordance with guidelines of the European Communities Council Directive of 24 November 1986 (86 609 EEC) and were accepted by the Ethics Committee for Animal Use of the University of Liège (protocol 86).

### Modeling

The model follows the common equation
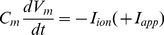
(1)where 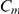 is the membrane capacitance, 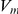 the membrane potential of the cell, 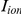 accounts for all the ionic currents and 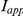 is any externally applied current. The fast dynamics include a sodium current 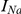 and a delayed-rectifier potassium current 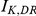. These currents are responsible for the creation of action potentials. The slow dynamics describe the fluctuations of 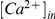. Calcium influx is mediated by a L-T-type calcium current 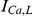, which is both voltage-gated and calcium-regulated, and the calcium is pumped out of the cell by calcium pumps, which generate an outward calcium current 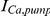. In order to reproduce *in vivo* conditions, the model can be subjected to excitatory synaptic inputs, which activate a synaptic current 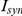. The background of this synaptic activity (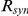) was modeled through stochastic waveforms. A calcium-activated potassium current 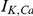 and a leak current 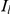 are also implemented in the model.

#### Equations and parameters

The equation giving the membrane potential variations over time is as follows:
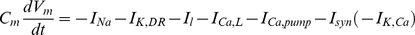
(2)


All the ionic currents equations follow a Hodgkin-Huxley scheme [31]. Each voltage-dependent channel expresses activation gates, whereas inactivation gates are only present in the equation for the sodium current. Control of L-type calcium channels by 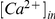 is incorporated through factor 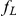. The currents are thus implemented as follows:
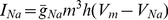
(3)

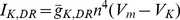
(4)

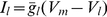
(5)

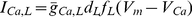
(6)

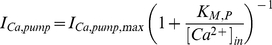
(7)

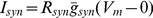
(8)

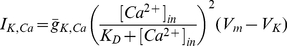
(9)with
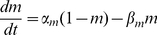
(10)

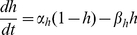
(11)

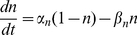
(12)

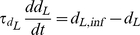
(13)

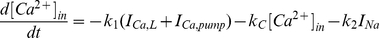
(14)where
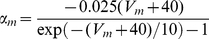
(15)

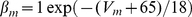
(16)

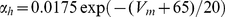
(17)

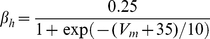
(18)

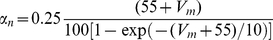
(19)

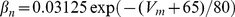
(20)

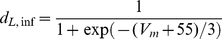
(21)

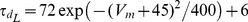
(22)

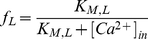
(23)


The values of the parameters used for the simulations are given in Table 2. An inhibition of the SK current was performed by reducing the value of its conductance to 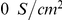. In order to ensure the simplicity of our model, we chose to use the parameters of the original Hodgkin-Huxley model [31] for the sodium, potassium and leak currents. These parameters were then slightly modified to fit the firing frequency of DA neurons. However, the mechanisms highlighted are robust to parameter variations, and are still present using the original values. The parameters for the L-type calcium current were taken from [14]. The only parameter that had to be tuned was the relative difference in timescales between the fast (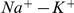) and slow (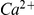) dynamics (namely the value of 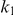), to ensure a spontaneous single-spike activity rather than endogenous bursting. In the equation describing the fluctuations of 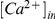, the term 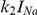 accounts for any voltage-dependent calcium entry unrelated to L-type calcium channels, to ensure a minimal calcium oscillation even when these channels are fully inhibited. In the detailed model [14], this calcium entry is provided by other voltage-dependent calcium channels.

**Table 2:** Parameter values.

Parameter	Value
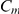	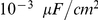
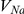	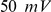
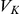	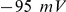
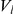	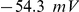
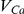	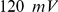
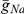	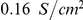
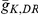	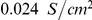
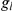	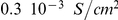
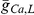	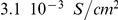
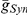	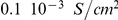
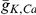	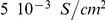
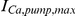	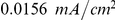
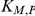	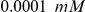
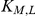	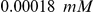
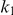	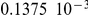
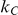	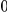
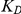	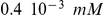
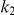	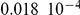

### Analyses

Simulations were performed using the Neuron software, which is freely available for download at http://www.neuron.yale.edu. Analyses were performed with Matlab 7.4.0.

#### Bifurcation diagram and variations of calcium thresholds

Bifurcation diagrams were drawn both using the graphical Matlab package matcont (which is freely available for download at http://www.matcont.ugent.be/) and the results of simulations.

### *In vitro* extracellular recordings

#### Housing

Adult (200–250 g) male Wistar rats were housed in groups of three or four, supplied with food and water ad libitum, and maintained on a 12 hour light/dark cycle.

#### Tissue preparation

Rats were anesthetized with chloral hydrate (400 mg/kg, i.p) and decapitated. The brain was extracted out of the skull in less than 1 min and cooled in ice-cold (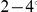
C) artificial cerebrospinal fluid (ACSF) of the following composition (in mM): 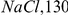; 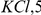; 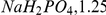; 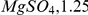; 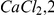; 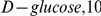; 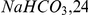; saturated with 95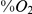 and 5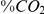 (pH 7.4).

Horizontal slices (400 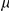
m) containing the midbrain were cut in cold ACSF in a vibratome and transferred to a small beaker containing ACSF at 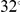
C. After 10–30 min slices were transferred to the recording chamber (volume 0.5 ml) in which they were completely submerged and superfused by ACSF at a rate of 2.5
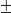
0.5 ml/min (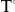: 34.5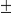 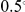
C). The tissue was held in position by small platinum weights.

#### Action potential recordings and identification of neurons

Action potentials (amplitude: 200 to 1000 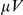) were recorded extracellularly using borosilicate capillaries (model 1403516, Hilgenberg, Malsfeld, Germany) which had been pulled on a Narishige (PE-2) vertical puller. The tip resistance was 5–20 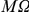. They were passed through an impedance adapter and amplified one thousand times with a homemade amplifier. They were displayed on an oscilloscope and fed to an analog digital interface (CED 1401) connected to a computer. Data were collected with the use of the Spike 2 software (version 4.23, Cambridge Electronic Design, Cambridge, UK). Use of an appropriate template ensured that the activity of only one cell was recorded.

Electrophysiological criteria were used in order to identify DA neurons. In vitro, these neurons exhibit a very regular firing pattern and long (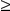
2.5 ms), triphasic spikes, often displaying a prominent notch in the initial positive rising phase. They have a slow firing rate comprised between 0.5 and 5 Hz. A large number of previous experiments in our laboratory have shown that the firing of these cells is inhibited by nanomolar concentrations of D2 agonists such as quinpirole and BHT920 [32]. This was verified again in this study in the absence of synaptic blockers (Supplementary Fig. 2B). Neurons meeting these criteria were selected for recording.

Only one cell was studied per slice. All recordings were made in the substantia nigra pars compacta area. Drugs were applied by superfusion at known concentrations using three-way taps. Each concentration was applied for at least 10 min to ensure that the drug concentration reached equilibrium in the tissue. Drug effects were quantified by calculating the mean firing frequency over the 5 last minutes of the control period and over the last minute during which the drug was superfused. Synaptic blockers were superfused throughout all experiments unless stated otherwise. Neurons whose firing rate varied by more than 5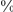 (average of 1 min) during the control period were discarded.

#### Experimental protocol

The activity of DA neurons was recorded in control conditions for 5 minutes. Synaptic blockers (10 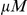 CNQX, 1 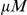 MK801, 10 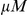 SR95531, 1 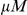 sulpiride and 1 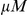 CGP55845 to block AMPA, NMDA, 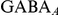, D2 and 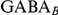 receptors, respectively) were then superfused for the remainder of the experiment. After at least 10 minutes, L-type calcium channels were blocked by bath application of 20 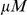 nifedipine for 10 minutes as well. When this compound completely blocked the firing of the cell, the experiment was stopped at this stage. Otherwise, nifedipine was washed out for 10 minutes. The sodium channel blocker tetrodotoxin (TTX) was then applied at 30 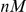 for 10 minutes. Again, if TTX completely blocked the firing of the cell, the experiment was stopped. Otherwise, TTX and nifedipine were co-applied for the next 10 minutes. After this period, TTX and nifedipine were washed out.

#### Drugs

The sources of the drugs used were as follows. CGP55845, CNQX, MK801, TTX and Nifedipine were obtained from Tocris Cookson (Bristol, UK). SR95531 was obtained from Sigma (St Louis, MO, USA). Sulpiride was a gift from Sanofi-Aventis.

#### Statistical analysis

Experimental data were analyzed with a global ANOVA test for correlated values, followed by Tukey's post-hoc tests for comparisons between groups.

## Supporting Information

Figure S1 Cooperation between sodium and calcium channels in the generation of spontaneous activity in the detailed model. The center panel shows the type of pacemaker activity according to the sodium and L-type calcium conductances. The white zone represent the couples of conductances which results to a spontaneous hyperpolarization of the cell and the dark blue zone account for pacemaking. Each insert shows the behavior of the model in control condition and during a blockade of L-type calcium channels or sodium channels for a particular set of conductances, respectively. The pacemaker behavior of the model strongly relies on the values of both the sodium and the L-type calcium conductances.
(EPS)

Figure S2Effect of synaptic blockers and a D2 receptor agonist on the firing rate of SNc DA neurons in brain slices. (A) Mean firing frequency (samples of 2 minutes) of the recorded cells over time (mean 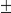 sem, n = 4). (B) Mean firing frequency (samples of 30 seconds) of the recorded cells over time (mean 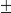 sem, n = 6). The application of synaptic blockers induces an increase in the firing rate which remains stable for at least one hour. The application of the D2 receptor agonist BHT 920 (100 nM) [32] strongly reduces the firing rate of the recorded cells.(EPS)

Figure S3 I-V curves of various depolarizing currents in the two models. I-V curves of sodium channels (in red) and of L-type calcium channels (in black) of the minimal model (A) and of the detailed model (B). For the detailed model, I-V curves of N-type (in blue) and T-type (in dotted black) calcium channels are also plotted. The only current which has a significantly different half-activation potential as compared to the L-type calcium current is the T-type calcium current.
(EPS)

Figure S4Analysis of the slow oscillatory potentials of the minimal model. (A) Variations of the membrane potential (top) and of the intracellular calcium concentration (bottom) over time. (B) Sketch of the bifurcation diagram of the minimal model, with 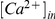 as the bifurcation parameter. The gray part corresponds to negative values of 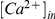, which are non physiological. 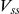 denotes the steady-state curve for each value of the bifurcation parameters. The dotted part of 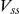 shows its unstable part. SN denotes a saddle-node bifurcation. Trajectories of the membrane potentials are plotted in red.(EPS)

Figure S5 Comparison of the calcium dynamics during pacemaking and slow oscillatory potentials. (A and B) Evolution of membrane potential (top) and calcium oscillations (bottom) over time in control conditions and during a sodium blockade, respectively. (C) Variations of intracellular calcium concentrations during both oscillatory patterns.
(EPS)

Figure S6Effect of sodium channel density on the response of the detailed model to calcium channel blockade. Response of the detailed model to an inhibition of all calcium channels for two slightly different values of sodium channel density. (A and B) Variations of the membrane potential over time in control conditions (left) and after the blockade of all calcium channels (right) for two different sodium conductances. Note how dramatically the value of 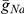 influences the effect of calcium channel blockade.(EPS)
